# Connecting the Global Climate Change and Public Health Agendas

**DOI:** 10.1371/journal.pmed.1001227

**Published:** 2012-06-05

**Authors:** Maria Nilsson, Birgitta Evengård, Rainer Sauerborn, Peter Byass

**Affiliations:** 1 Umeå Centre for Global Health Research, Department of Public Health and Clinical Medicine, Umeå University, Umeå, Sweden; 2 Institute of Public Health, Heidelberg University Medical School, Heidelberg, Germany; 3 MRC/Wits Rural Public Health and Health Transitions Research Unit (Agincourt), School of Public Health, Faculty of Health Sciences, University of the Witwatersrand, Johannesburg, South Africa

## Abstract

Peter Byass and colleagues urge public health professionals to strengthen their response and develop actions to bring health and climate co-benefits.

**Summary Points**

- Climate change is a public health problem. Evidence from many sectors shows substantial health impacts of climate change, particularly for the most vulnerable: the poorest, the youngest, and the oldest.
- Human health and climate change are closely connected. Within the global United Nations (UN) process, health is seen as the most direct component linking climate change and individual lives.
- Public health actions in relation to climate change are needed. Top-down advocacy on health and climate at the UN level needs to be mirrored by bottom-up public health actions that bring health and climate co-benefits.

## Why Is Climate Change a Public Health Problem?

As the dust settles on the November 2011 United Nations (UN) climate talks in Durban, it is opportune to reflect on the likely consequences of the proceedings for human health, and in particular what public health actions should be prioritised in response to the global climate situation.

Human beings live in a biosphere in which the transient forces we know as “weather” considerably influence lifestyle and behaviour. Over longer time periods, “weather” is interpreted into “climate”, which has longer-term effects on human health. Changes in established patterns of climate influence population health, affecting daily life, altering patterns of physical activity and food availability, and in some cases bringing direct physical harm. In whatever ways climate change unfolds over the coming decades, populations at the global extremes are likely to be among the worst affected. Slightly higher temperatures in the world's hottest regions could render living and working conditions marginal or untenable [1]. In the Arctic region, although absolute temperatures will remain cool, relatively small changes in climate are likely to result in major public health consequences such as changing patterns of infectious diseases or disruption to traditional food supplies [2]. A specific Arctic region side-event in Durban reflected on an Arctic Council report that concluded the last six years have been the warmest ever period in the Arctic [3], driving an increased sense of urgency to act on short-lived climate forcers (SLCFs). SLCFs such as atmospheric soot have a relatively short life in the atmosphere, but influence climate powerfully, so possible reduction strategies can lead to relatively short-term climate benefits compared with, for example, CO_2_ emission reduction. Concern is also growing about a lack of food security among northern indigenous peoples as a consequence of climate change [4]. Clearly, these problems share no universal solution applicable to all countries and regions. In between the climatic extremes, people are already experiencing the effects of “rare” weather events becoming increasingly common [5], often with injurious consequences.

## How Does Health Fit into the Overall Climate Change Agenda?

What the world came to know as “the Durban Climate meeting” was more properly known as The Seventeenth Conference of the Parties to the United Nations Framework Convention on Climate Change (COP17, UNFCCC). The UNFCCC requires Parties to “Take climate change considerations into account … with a view to minimizing adverse effects … on public health” [6]. The most important Parties involved are UN member states. In addition, there are numerous other organisations accredited to the UNFCCC, contributing science, influencing policy, and bringing world voices to the process; one such organisation is our Centre for Global Health Research at Umeå University (http://www.globalhealthresearch.net).

Given the UNFCCC's formal structure, and the huge number of people involved, unsurprisingly COP17 ended up in extra-time negotiations to hammer out consensus on a global way forward on emissions reductions, as was widely reported in the general media. The increasingly powerful alliance between European and developing countries, representing the overwhelming majority of the world's nations, was a hugely positive influence at the negotiating table. But in parallel with these big concerns, our overall impression was that health issues and specific health events are acquiring a more solid base within the UNFCCC process, in contrast to earlier stages when it sometimes seemed that climate threats to species other than *Homo sapiens* predominated. Indeed, the international public health community is gaining traction on connecting climate and health, as reflected in several substantial publications in recent years [7],[8]. Alongside the main agenda in Durban, a Climate and Health Summit brought together leading health protagonists from more than 40 countries, the first such major event linking climate and health issues [9]. Keynote speakers included South African Minister of Health Dr. Aaron Motsoaledi and Prof. Hugh Montgomery of University College London, who underscored the urgency of the problem by asserting that “If we plan to write a prescription in eight years' time, we might instead find ourselves writing a death sentence.”

## How Should the International Public Health Community Act on Climate?

**Figure 1:**
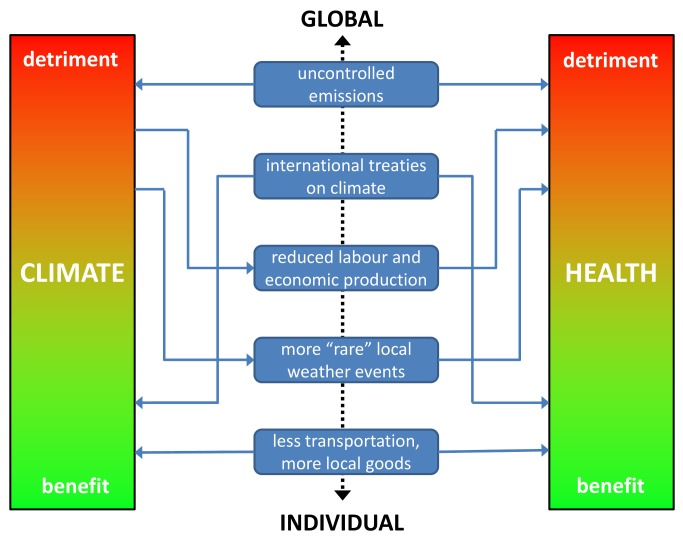
Schematic of inter-relationships between climate and health, with examples of determinants on a global to individual scale.

The complex interactions between human behaviour, climate, politics, and health on a global basis mean that the public health community must not confine itself to situational analyses and prognoses, but move to actions. Possible climate changes constitute a public health crisis at least as wide-ranging as the effects of tobacco on health, and there may be lessons to be drawn from experiences with anti-tobacco initiatives [10]. Figure 1 shows some examples of inter-relationships between climate and health, mediated by determinants that range from the global to the individual.

Within the climate change context it is possible to identify a number of co-benefits encompassing climate and health. Encouraging people to walk and cycle rather than using motorised transport, and to eat healthier, locally produced foodstuffs, are clear examples that can bring both individual health benefits and reduced climate impact. Many organisations represented in Durban were advocating specific practical initiatives along these lines. However, as yet there seems to be a lack of coherence in terms of clear public health messages about climate aimed at populations in general. There remains, therefore, a major challenge for the public health community to identify best individual and local strategies, develop corresponding messages, and enable people to adopt climate-friendly lifestyle changes that are also healthy—so-called health and climate co-benefits.

Moving up from the individual to the corporate and societal levels reveals the need for further actions. For example, the health sector institutionally needs to carefully consider, and reduce, its climate change footprint [11]. In general, individuals cannot regulate their lives in terms of carbon footprint, for example, in a way that is completely independent of the societies in which they live [12]. Lessons can be drawn from initiatives such as the healthy cities movement, which seeks to build social change collectively within local communities, and so brings distinct possibilities for community-level actions on climate change.

At the global level, the need for consensus actions on climate that only governments can make is equally important, and public health voices must be heard on health-related issues in those circles, including lending support and influence for legislation, regulatory action, or other reform designed to address climate and environmental concerns. It is becoming increasingly clear that maintaining a sustainable and healthy climate is something that can only be achieved by means of a concerted global effort, including large-scale and small-scale actions, in which the public health community must play an active part.

## Conclusion

Thin ice is an increasing reality in the Arctic, as well as a metaphor for impending disaster. The conclusion of negotiations in Durban brings some encouragement, but still leaves the world, and not just the Arctic, on thin ice. We hope that the continuing UNFCCC process will nevertheless lead to an improving global prognosis—to which the public health community must contribute by effectively promoting health and climate co-benefits.

## Abbreviations

SLCF: short-lived climate forcer
UN: United Nations
UNFCCC: United Nations Framework Convention on Climate Change
